# Health inequalities in the management of chronic hepatitis B virus infection in patients from sub-Saharan Africa in high-income countries

**DOI:** 10.1016/j.jhepr.2022.100623

**Published:** 2022-11-04

**Authors:** Tim Mitchell, Jeremy S. Nayagam, Geoffrey Dusheiko, Kosh Agarwal

**Affiliations:** 1 Institute of Liver Studies, King’s College Hospital, London, United Kingdom; 2 Gastroenterology and Hepatology Department, Royal Perth Hospital, Perth, Australia; 3 Department of Inflammation Biology, King’s College London, London, UK; 4 University College London Medical School, London, UK

**Keywords:** Healthcare engagement, elimination, clinical trials, hepatocellular carcinoma, migrant health, HBV, hepatitis B virus, HCC, hepatocellular carcinoma, MAFLD, metabolic dysfunction-associated fatty liver disease, SSA, sub-Saharan Africa, TAF, tenofovir alafenamide, TDF, tenofovir disoproxil fumarate, TE, transient elastography, WHO, World Health Organisation

## Abstract

Chronic hepatitis B virus disproportionately affects migrant communities in high-income countries, reflecting increased migration from sub-Saharan Africa. Chronic hepatitis B virus is endemic in sub-Saharan Africa, yet the natural history of chronic infection experienced by patients remains incompletely understood, with evidence of variability across genotypes and regions within sub-Saharan Africa. Clinical guidelines recommending treatment thresholds are not specific to sub-Saharan African patients and are based on natural history studies from Western Pacific Asian countries. Access to standard of care treatment is available for sub-Saharan African people with chronic hepatitis B virus infection in high-income countries; however, the evidence base for these treatments was not established in this cohort and areas of uncertainty remain, particularly regarding HCC surveillance and treatment discontinuation. Participation in phase III clinical trials for chronic hepatitis B therapies is almost non-existent amongst sub-Saharan African patients, even when residing in high-income countries that participate in multicentre trials. Engagement with sub-Saharan African patients with chronic hepatitis B in high-income countries is challenging because of the stigma associated with the diagnosis, absence of routine screening systems and the complexities involved in navigating the healthcare system. Nonetheless, improved engagement is critical if we are to achieve global hepatitis B virus elimination.

**Key points**

• In high-income countries, chronic hepatitis B virus (HBV) infection is most prevalent in migrant communities from endemic areas, such as sub-Saharan Africa.
• The majority of evidence for chronic HBV infection management was derived from Western Pacific Asian populations.
• Despite acknowledgement that the natural history of chronic HBV infection in a sub-Saharan African population is different, guidelines developed in high-income countries do not account for these differences.
• Enrolment of sub-Saharan African patients in major chronic HBV infection clinical trials run in high-income countries is poor, preventing an applicable evidence base from being developed.
• Increased engagement with migrant communities in high-income countries is critical to the worldwide goal of HBV elimination by 2030.

## Introduction

Chronic hepatitis B virus (HBV) infection affects approximately 300 million people worldwide.^1^ It is estimated that one patient dies every 30 seconds from their illness.^2^ In response to this global disease, the World Health Organisation (WHO) is targeting the elimination of viral hepatitis as a public health threat by 2030.^2^ Progress so far has not been consistent across all regions and must be accelerated if these targets are to be met.^1^ Sub-Saharan Africa (SSA) has the second highest prevalence of chronic HBV in the world, at 6.5% – it is estimated that approximately 80 million people are living with chronic HBV in the region.^1^^,^^2^ The high rates of chronic infection are largely determined by early childhood infection including residual mother to child transmission. Chronic HBV is responsible for 41% of cirrhosis cases in SSA compared with only 13% in Europe.^3^ Progressive disease is common in SSA, where 90,000 people per year die from cirrhosis and hepatocellular carcinoma (HCC).^4^ Despite reductions in the cost of antiviral treatment, efforts to eliminate HBV in SSA have historically been hampered by a combination of low awareness, low rates of diagnosis, impaired access to nucleic acid testing, inadequate infrastructure and healthcare resources, as well as lower vaccination uptake and access to antiviral treatment.^4^^,^^5^

In high-income countries, HBV disproportionally affects migrant populations from endemic areas, who contribute a high proportion of cases of chronic HBV.^6^ For example, migrants contribute 25% of chronic HBV cases in European Economic Area countries whilst only representing 5-10% of the total population.^6^ The disparity is even higher in the UK where 6% of the population contribute 72% of chronic HBV cases.^6^ A significant proportion of migrants now originate from endemic countries within SSA rather than Asia or Eastern Europe.^7^^,^^8^ Thus, a recent report indicated that in London, individuals identifying as Black are almost five times more likely to test positive for HBV than Caucasian patients.^9^ Despite increasing migration from regions without established HBV detection and treatment programmes, routine screening of immigrants is still not performed in the UK.[10], [11], [12]

The large African diaspora exposes deficiencies and provides an opportunity for improved care in chronic HBV. Guidelines on the management of chronic HBV from major hepatology or infectious disease societies are not based on evidence obtained in patients from SSA, instead recommending treatment and surveillance approaches established from cohort studies in Western Pacific Asian and Caucasian patients.^13^ The COVID-19 syndemic has highlighted the impact societal determinants of health have on the outcomes of disease, particularly in Black and minority ethnic groups.^14^ Failure to develop a nuanced, evidenced-based approach to chronic HBV in populations from SSA will prevent the realisation of the WHO’s ambitious viral hepatitis elimination target and may lead to a similar chronic HBV syndemic in high-income countries, driven by the social stigmas and healthcare inequity experienced by migrant populations.^15^^,^^16^

In this Public Health article, we will review the evidence base for chronic HBV management in high-income countries and discuss the evidence gaps that require immediate attention regarding patients from SSA, particularly HCC surveillance, involvement in clinical trials and linkage to care.

## HCC surveillance

Chronic HBV is an important risk factor for the development of HCC although there is geographic variation in the risk of HCC associated with chronic HBV, which is seen across the different viral phenotypes.^17^ The 5-year cumulative incidences of HCC for low viral replicators, chronic HBV without cirrhosis and chronic HBV with compensated cirrhosis in East Asian populations are 1%, 3% and 17%, respectively, which are higher than the 0.1%, 1% and 10% observed in Western Europe and the United States.^18^ It is suggested that this is related to risk-factor exposure and age of acquisition more than genetics.^19^ Despite only sparse data in patients from SSA, guidelines highlight ethnicity as an important factor in HCC development.^17^

A specific risk factor pertinent to SSA is aflatoxin B1 (AFB1), a mycotoxin synthesized by *Aspergillus flavus* and *Aspergillus parasiticus,* which is a potent hepatocarcinogen.^20^ Chronic AFB1 exposure confers an increased risk of developing HCC.^20^ The signature mutation induced by aflatoxins is the mutation at codon 249 in the tumour suppressor gene TP53 (R249S).^20^ Epidemiologic studies have demonstrated a strong correlation between dietary intake of AFB1, TP53 mutations and the incidence of HCC, in HBV-infected individuals in SSA and elsewhere.^21^^,^^22^ Maize, and other staple foodstuffs including ground nuts, are susceptible to contamination by improper drying in tropical and sub-tropical climates, conferring chronic hazardous exposure.^20^ AFB1 levels exceeding WHO safety levels have been documented in several countries in SSA.^20^

The major international liver societies have published guidelines on surveillance for HCC in individuals with chronic HBV (Table 1) that incorporate 6-monthly ultrasound with or without alpha-fetoprotein (AFP) measurement. Although there is a consensus that all patients with cirrhosis should be entered into a surveillance programme, noting this is necessary to diagnose early HCC that may be treated with curative intent, the wide variations in recommendations for patients from SSA reflect, in part, the lack of data for this population. The European Association for the Study of the Liver acknowledges that despite increasing migration to Europe of individuals from endemic regions, the overall impact on HCC incidence has not been investigated.^17^ Locally formulated risk stratification scores need to be developed and validated to incorporate known risk factors and diminish the burden of surveillance of low-risk populations.^17^^,^^23^^,^^24^

**Table 1:** Society recommendations for HCC surveillance in African patients with chronic HBV.

Society	Recommendation
AASLD^19^	African males aged >40 years
EASL^17^	No recommendation
APASL^23^	All African patients aged >20 years

AASLD, American Association for the Study of Liver Diseases; APASL, Asia-Pacific Association for the Study of the Liver; EASL, European Association for the Study of the Liver; HBV, hepatitis B virus.

Scoring systems have been developed to stratify the risk of HCC in individuals with chronic HBV, and to expedite and improve HCC surveillance programmes. GAG-HCC, CU-HCC and REACH-B were derived and validated in Western Pacific Asian populations.[25], [26], [27] The derivation cohorts for GAG-HCC and CU-HCC included individuals with cirrhosis; thus, these scores weigh the risk associated with cirrhosis in calculations.^25^^,^^26^ REACH-B was derived from a non-cirrhotic cohort, and therefore does not include cirrhosis as a variable.^27^ All scores include a weighting for HBV DNA; however, in Western Pacific Asian patients on long-term nucleos(t)ide analogues (NAs), replacing HBV DNA with transient elastography (TE) in the mREACH-B score improved accuracy.^28^^,^^29^ Future risk models will likely be biomarker driven, for example GALAD, which incorporates AFP, AFP-L3 and des-gamma-carboxy-prothrombin into a model with age and sex.^30^

There is an important need to validate these scores in different ethnic cohorts, in whom differing modes of transmission, age of acquisition, HBV genotypes, and past or current environmental risk factors could alter risk stratification.^26^^,^^27^ A larger proportion of Western Pacific Asian patients acquired HBV through perinatal transmission, are infected with genotype B or C, and consequently have higher rates of HBeAg-positive infection and disease.^31^ Individuals born in SSA are more likely to have acquired HBV via horizontal or parenteral transmission in childhood and there are higher rates of anti-HBe-positive infection.^32^ There are regional differences in the prevalence of HBeAg positivity within Africa, with this group more prevalent in West and Central Africa where genotype E is predominant.^20^ Genotype A1 is most common in Eastern and Southern Africa and chronic infection with genotype A is associated with a higher relative risk of HCC development than non-A genotypes.^33^

SSA faces an increasing burden from harmful alcohol consumption, although the relative contribution of alcohol to HCC development differs by region and cultural or religious beliefs.^20^ Metabolic dysfunction-associated fatty liver disease (MAFLD) is increasing in prevalence, adding an attendant HCC risk.^20^ The rising prevalence in SSA is of concern. It will be important to raise public awareness regarding MAFLD, to offset the effects of this comorbidity in Africans.^34^ Untreated HIV and HBV or HCV coinfection increase the risk of progressive disease and HCC.^20^ Critically, a scale up of anti-retroviral treatment of HBV-HIV disease with regimens including tenofovir or tenofovir alafenamide, active against hepatitis B, will reduce the risk of HCC. Similarly, the diagnosis and cure of hepatitis C will be the most effective secondary preventive measure, but both measures will necessitate improving awareness and providing effective linkage to antiviral treatment. Within SSA, a coordinated agronomic approach to reduce crops’ susceptibility to AFB1 needs to be considered. These measures include prevention of contamination with adequate irrigation and use of fungicides, sun-drying on cloth and not soil, and hand-sorting to remove mouldy crops.^20^ Aflasafe^R^, a biopesticide applied to maize to displace aflatoxin-synthesising fungal strains has been developed to reduce toxin-producing aflatoxin contamination of crops.^20^ PACA (The Partnership for Aflatoxin Control in Africa) is a group that aims to mitigate aflatoxin exposure. Primary prevention of all the ancillary factors that increase the risk of HCC in HBV-infected sub-Saharan Africans living in the African diaspora will be key to reducing the ultimate incidence of HCC in these cohorts.

It is striking that despite the acknowledgement that individualised risk calculators are needed for patients of different ethnicities and from different geographical regions, there have been no generalisable prescriptive guidelines derived or validated in individuals from SSA with chronic HBV. This brings into question our ability to formulate an appropriate risk-stratified HCC surveillance system for individuals from SSA with chronic HBV.

## Antiviral treatment

The main goals of therapy for chronic HBV are to improve survival and quality of life by preventing disease progression and complications of end-stage liver disease, such as HCC.^17^ Long-term treatment with NAs is associated with a histological improvement in liver fibrosis and a reduction in the need for liver transplantation for decompensation.^17^^,^^35^ Although the risk is reduced by effective NA therapy, HCC can still develop. The residual risks of HCC have been extensively documented in non-sub-Saharan African populations.

In large cohorts of Western Pacific Asian patients treated with NAs, significant reductions in the development of HCC have been observed both for those with and without cirrhosis.^36^ Those with pre-existing cirrhosis have a 5.49-fold greater risk of HCC development than those without cirrhosis at baseline.^37^ The risk reduction from NAs is predicted to be 73% in individuals without cirrhosis and 77% in those with cirrhosis.^36^

A European study including mainly Caucasian and Asian patients, identified a low but significant rate of HCC in those on long-term NAs, despite virological response.^38^ A study of Caucasians treated with NAs (entecavir or lamivudine) identified a higher risk of HCC in those with cirrhosis, with a 5-year incidence of 9% compared to 1% in those without cirrhosis.^39^ A large study of Caucasian patients on long-term NAs, who were followed from year 5 to year 10 after commencing NAs, identified no change in the yearly incidence of HCC across the first and second 5 years in individuals without cirrhosis (0.5% *vs.* 0.5%), but a significant reduction in yearly incidence in the second 5 years in those with cirrhosis (3.2% *vs*. 1.6%).^40^

An area of debate is which NA, tenofovir or entecavir, is associated with a bigger reduction in HCC risk. Although a recent systematic review and meta-analysis concluded that tenofovir is associated with a lower risk of HCC than entecavir, conflicting data has emerged from a large study which reported no difference between agents when adjusting for factors including age and cirrhosis, which was echoed in a recent meta-analysis with propensity matched individuals.^41^^,^^42^ The majority of this data is from Western Pacific Asian populations and has not been reported in other ethnicities. As yet, there is no data in patients from SSA to inform guidelines or clinical management.

Highly potent and cost-effective oral antiviral treatments are widely available for individuals with chronic HBV living in high-income countries.^17^ A reduction in the risk of HCC has been observed in those with and without cirrhosis, clearly demonstrating clinical endpoint benefit.^17^ Although maintenance suppressive treatment is required, treatment costs with either generic entecavir or tenofovir are low – less than £150 per year. Guidance for physicians, highlighting the optimal timing of treatment to prevent complications such as cirrhosis and HCC, has been published by international liver societies.^17^^,^^19^^,^^23^ The guidelines describe the classic four phases of chronic HBV infection and propose treatments based on natural history studies such as REVEAL-HBV, from data ascertained in Taiwan.^43^

However, current indications for treatment are not based on the molecular biology, natural history and morbidity of chronic HBV in populations from SSA. Although the prevalence of chronic HBV-associated HCC in west, central, east and south SSA has been detailed, regional prospective natural history studies have not been performed in large, endemic chronic HBV populations in SSA. Despite the authors encouraging studies to take place in other populations at high risk of HCC, such as African Americans, there are few data on how effective long-term maintenance therapy with NAs is at preventing HCC in patients from SSA.^36^ The majority of the data to frame guidelines has been reported separately in either Western Pacific Asian or Caucasian cohorts, and it is not clear how generalisable these findings are. Nonetheless long-term prospective studies comparing antiviral treatment to no treatment would now be ethically impossible, given the advent of effective and affordable antiviral treatments.

Studies to confirm a reduction in liver-related morbidity after prolonged NA therapy in individuals from SSA are lacking. The underlying lack of validation of current guidelines in these patients potentially justifies the need for specific guidelines to be developed in this population, while examining the utility and relevance of current guidelines.^44^ Leaving patients with chronic HBV and evidence of hepatic inflammation untreated for a prolonged period is unethical. Therefore, selection of patients from SSA for treatment will have to be reasonably based on guidelines derived from data in other populations until (and if) definite evidence teaches us otherwise. The existing international guidelines on treatment of hepatitis B rely on a clinical diagnosis and a combination of abnormal serum aminotransferase concentrations, and HBV DNA levels.^17^ The widespread lack of access to HBV DNA measurement has resulted in secondary but poorly predictive WHO guidelines in areas where HBV DNA testing is unavailable.^13^ These guidelines are reliant on non-invasive scores that favour treatment of advanced disease rather than treatment to prevent the onset of cirrhosis and sequelae of cirrhosis.^13^^,^^45^ Other scores, not including HBV DNA, such as TREAT-B, are being validated in SSA, but guidelines which do not include HBV DNA measurement are seemingly unnecessary in high-income countries.

The relatively low replicative state in anti-HBe-positive patients from SSA alongside the dichotomous risk of HCC in the region imply that thresholds for treatment should potentially be different in individuals from SSA. Studies to confirm a reduction in liver-related morbidity after prolonged NA therapy will be difficult to perform prospectively. Until specific evidence and guidelines can be substantiated, treatment should be offered to patients from SSA who would otherwise meet established treatment criteria.^17^ Earlier initiation of treatment for patients outside this criteria with additional risk factors for HCC development (*e.g.* increased age, MAFLD) could also be considered. Other markers including serum pregenomic RNA and hepatitis B core-related antigen require further study, as does the necessity to widen treatment indications in populations from SSA.

TE is readily available in the UK to rule in minimal fibrosis and defer treatment in patients with a serum HBV DNA concentration consistently <2,000 IU/ml and normal serum aminotransferases.^17^ TE can also identify advanced fibrosis or cirrhosis.^17^ A composite guideline utilising serum markers, serum aminotransferases and a single TE measurement necessitates the fewest investigations and follow-up appointments in tertiary centres, making it suitable for migrant populations and sustainable in a high-income healthcare system.^46^ There are moves to place non-invasive technologies for fibrosis staging in more accessible community settings.

Currently recommended treatments for chronic HBV include entecavir and tenofovir disoproxil fumarate (TDF), with an emerging role for tenofovir alafenamide (TAF).^17^ Guidelines point to excellent response rates in randomised-controlled trials; long-term meta-analyses demonstrate a significant reduction in progression to cirrhosis, HCC risk and mortality.^17^ Nonetheless, on average, patients from SSA comprise <5% of the study cohorts of the major clinical trials, with an expected lack of representation of the most common African genotypes A1 and E. Long-term registry studies assessing outcomes and safety are similarly comprised almost exclusively of Western Pacific Asian patients.^18^

Although limited data from studies in SSA populations suggest good viral suppression, the follow-up duration of these studies is relatively short.^47^^,^^48^ Current data suggest that persistently undetectable HBV DNA in serum can be achieved in patients from SSA. However, similar to Caucasian and Asian patients, loss of HBsAg is rare, as current NA treatments do not eradicate cccDNA. Current studies of functional cure are being undertaken worldwide and it is important that patients from SSA are included in these studies to determine the effect on HBsAg in predominantly anti-HBe-positive patients. This is especially important as patients from SSA could be considered to be at higher risk of the most widely reported potential adverse effects of long-term TDF – renal impairment and bone disease.

Renal function has been shown to decline faster in African-American people without known chronic kidney disease than in Caucasian populations.^49^ In the UK, hypertension and diabetes – two major risk factors for renal disease – have a significantly higher prevalence in patients from SSA compared with the rest of the population.^50^ Data from HIV/HBV-coinfected patients from SSA has confirmed a statistically significant decline in renal function in those on TDF.^51^ Switching HIV/HBV-coinfected patients to TAF is associated with improved estimated glomerular filtration rate and a reduction in proteinuria.^52^ Conversely, a recent publication from HIV/HBV-coinfected individuals in the USA, including 40% of Black ethnicity, reported significant weight gain early after switching from TDF to TAF.^53^ This could have a detrimental impact on the management of metabolic risk factors such as diabetes, hypertension and coexistent fatty liver disease.

Chronic HBV has been shown to be associated with an increased risk of hip fracture in African American patients, before treatment has even been commenced.^54^ Vitamin D deficiency is significantly more common in migrants to Europe from SSA compared to East Asian migrants and may further contribute to the risk of bone-related adverse effects.^55^ Resistance may be under-reported in patients from SSA as there is relatively little data to predict treatment resistance in HBV-monoinfected patients receiving either entecavir or tenofovir. Amino acid substitutions in the reverse transcriptase associated with reduced tenofovir sensitivity have been reported; although there is a high genetic barrier to selection of tenofovir resistance, a prior history of lamivudine and adefovir will increase the risk of resistance. There are few studies on adherence to therapy and virologic breakthrough in patients from SSA.^56^

Despite higher response rates observed in genotype A patients with high ALT and low HBV DNA levels, pegylated interferon alpha remains an accepted, but uncommonly, utilised treatment in the UK.^57^ Although the side effect profile is significant, the limited duration of treatment may appeal to a younger migrant population who are averse to the stigma associated with life-long treatment.

## Role for NA withdrawal

Curtailing NA treatment is currently being appraised in anti-HBe-positive patients.^17^ The potential benefits of NA withdrawal include HBsAg seroclearance, as well as the avoidance of potential long-term medication use and related side effects.^17^ The predominant clinical concern is the risk of precipitating acute liver failure.^58^ It is also not known whether increased HBV replication following NA withdrawal could result in an increased risk of HCC.^59^

The initial study included primarily genotype D patients from Greece.^60^ Subsequent studies have included both Western Pacific Asian and Caucasian patients. Rates of HBsAg clearance have varied widely, but recent randomised controlled trials including the FINITE and STOP-NUC trial suggest that between 10-19% of individuals may lose HBsAg after NA withdrawal.^59^^,^^61^ Unfortunately 50-60% relapse 12-36 months after cessation.^62^ Provocation of a “flare”, the risk of a clinical or virological relapse, severe hepatitis, and a poorly understood primed immune response following cessation of treatment ensures that predicting safe discontinuation remains a challenge.^63^

The likelihood of HBsAg clearance is restricted to those with a HBsAg concentration of <1,000 IU/ml, or lower, at the time of withdrawal.^64^ Genotype may be an important determinant of HBsAg loss.^65^ There are however no representative studies of SSA cohorts with genotype A1^66^ A systematic review from 2016 did not include a single African patient.^62^ Features that have been associated with surface antigen loss (anti-HBe positivity, lower viral load) are commonly seen in patients from SSA.^67^ Although the spontaneous rate of HBsAg loss appears similar to Western Pacific Asian populations (1% annually), the rate of surface antigen loss following NA withdrawal has not been studied in this population.^67^^,^^68^ This lack of data may prevent clinicians from offering a valid, potentially beneficial treatment to patients from SSA.

## Representation in clinical trials

Patients from SSA are poorly represented in multicentre therapeutic clinical trials for chronic HBV (Table 2). Despite the continent accounting for 25% of the global disease burden, less than 3% of clinical trials are performed in Africa.^69^ Suggested barriers to clinical trial implementation include financial capacity, regulatory and ethics review delays and complex logistical requirements.^70^ Nonetheless, with appropriate planning and determination, numerous high-quality clinical trials in other infectious diseases, including HIV, have been completed in SSA.^70^^,^^71^ There is no reason why this should not be achievable in chronic HBV (see Box 1).

**Table 2:** Summary of Black ethnicity and genotype involvement in major chronic HBV trials.

Study (first author, year)	Phase	Study population	Number	Black ethnicity	Genotype	Exclusion criteria
Lai *et al.* 1998^86^	III	China (HK, Taiwan, mainland China)	358	0 (0%)	Not specified	
Hadziyannis *et al.* 2003^87^	III	Canada, Greece, Israel, France, Italy, Australia, Taiwan, Singapore	185	6 (3.2%)	Not specified	
Chang *et al.* 2006^88^	III	Europe (41 centres), North America (40), Asia (26), Australia (12), South America (18)	715	16 (2.2%)	A – 27%, B – 20%, C – 27%, D – 12%, E – not mentioned, F – 4.5%	
Marcellin *et al.* 2008^89^	III	Europe (59%), North America (24%), Australia/NZ (17%)	641	30 (4.7%)	A – 16%, B – 12%, C – 17%, D – 50%, E-H – 4%	
Buti *et al.* 2016^90^	III	Canada (11 sites), USA (14), UK (2), France (2), Italy (4), Poland (4), Romania (5), Russia (10), Spain (1), Turkey (5), Australia (5), NZ (1), India (10), Japan (11), HK (4), South Korea (10), Taiwan (5)	425	8 (1.9%)	A – 5%, B – 24%, C – 38%, D – 31%, E – 2%	
Chan *et al.* 2016^91^	III	East Asia (18% of patients), Europe (18%), North America (16%), Australia (2%), NZ (2%), India (13%)	873	10 (1.1%) Nb. categorized as “other”	A – 7%, B – 17%, C – 52%, D – 23%, E – <1%, F – <1%	
Bazinet *et al.* 2020^92^	II	Republic of Moldova	40	0 (0%)	A – 3 (7.5%), D – 37 (92.5%)	ANC <1,500 cells/mm^3^

Box 1 **Future research opportunities for HCC in patients from SSA with chronic HBV.**

**Figure:**
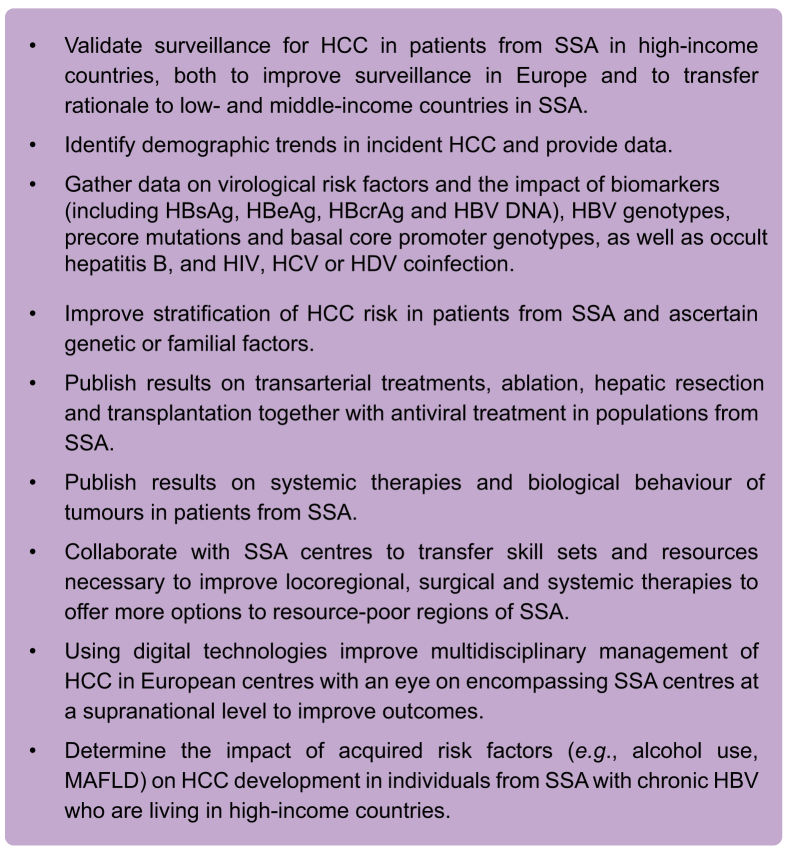

HBcrAg, hepatitis B core-related antigen; HBV, hepatitis B virus; HCC, hepatocellular carcinoma; MAFLD, metabolic dysfunction-associated fatty liver disease; SSA, sub-Saharan Africa.

Clinical trial involvement of patients from SSA living in high-income countries is also low. In high-income countries with large populations of patients from SSA, the low involvement rate may be due to systemic issues with blood parameters used as exclusion criteria and for grading adverse events. A study in healthy Eastern and Southern African patients who would be representative of those joining an AIDS clinical trial importantly demonstrated that this population had lower haemoglobin and neutrophil counts and higher bilirubin compared with United States reference intervals.^72^ Specifically, 14.9% had an elevated bilirubin and 9.6% low neutrophil count that would have counted as at least a grade 1 adverse event based on US National Institute of Allergy and Infectious Diseases Division of AIDS criteria.^72^

The upper limit of normal serum ALT varies based on the local population.^73^ Patients who have an ALT within the normal range at their hospital laboratory may be deemed to be outside the reference range and ineligible to enter trials if the central reference laboratory limit is based on a different ethnic population.^73^

Benign ethnic neutropenia (absolute neutrophil count <1.5 × 10^9^ cells/L) may also exclude patients from clinical trials, especially phase I trials.^74^^,^^75^ This condition has a prevalence of 6-8% but natural history studies have not shown any increased risk of infectious complications.^75^ In a review of prostate cancer trials, 41.4% of trials excluded patients with benign ethnic neutropaenia.^76^ Lowering the threshold for the absolute neutrophil count to 1.0 × 10^9^ cells/L would have improved trial eligibility without exposing patients to increased risk.^76^

A recent publication focusing on high-income countries with ongoing immigration found that HBV elimination will not be possible without the development of curative therapies.^77^ Systematic exclusion of patients from SSA from therapeutic clinical trials, regardless of whether patients are living in high- or low- and middle-income countries, limits diversity in clinical trials and ultimately disadvantages all. Pharmaceutical industry-run clinical trials must include more patients from SSA, to generate representative cohorts that more accurately reflect the worldwide burden of chronic HBV.

## Healthcare engagement and primary prevention

Elimination targets will not be met in high-income countries without an increase in awareness, diagnosis and treatment of migrant groups.^78^ In low endemicity countries, migrants account for the majority of chronic HBV cases, although case finding and linkage to care remains an issue.^79^ Despite demonstrated cost effectiveness and acceptability in the community, routine screening of migrants from moderate to high endemic countries, such as those comprising SSA, is not performed in the UK.^79^ Concerningly, few GP practices follow current guidelines on who to offer screening.^11^

Opportunistic programmes have shown success at identifying patients with chronic HBV and linking them to care in single UK centres.^80^ This also provides an opportunity to encourage vaccination among family members of identified patients and to prevent new infections among sexually active adolescents and adults. Screening of migrants arriving from countries with either poor diagnostic capabilities or HBV prevalence >1% appears cost-effective and should be undertaken as part of migration assessment.^79^ Recent modelling from Canada has reiterated that an aggressive screening and treatment strategy could decrease the incidence of HBV-related HCC by 26.1%, even if elimination in a country with high migration was not possible.^77^ Point of care testing utilising hepatitis B core-related antigen has been correlated with viral load, enabling identification of patients who meet treatment thresholds.^81^ When coupled with a simultaneous ALT elevation this would facilitate treatment initiation in mobile outreach settings.

Once identified, treatment uptake in individuals from SSA diagnosed with chronic HBV can be complicated by overarching cultural and religious values that vary between patients.^82^ A review from the United States identified several themes as barriers to chronic HBV treatment amongst migrants from SSA. These included a lack of knowledge or acceptance of chronic HBV as a disease, with some languages not having a word for hepatitis.^82^ Myths regarding the transmission of HBV and stigma surrounding the diagnosis may affect healthcare engagement, especially within small migrant communities.[82], [83], [84] A paradigm shift to preventative medicine can also be conceptually difficult to understand for patients who may have previously only attended the doctor with specific symptoms or problems to be resolved.^82^ As chronic HBV is often asymptomatic, it has been hypothesised that this may contribute to low rates of screening in high-income countries, emphasising the need for healthcare engagement.^82^

Additionally, chronic HBV presents a unique challenge in comparison to HIV or HCV as the determination for treatment is constantly under review.^78^ Rather than test and treat approaches in other infectious diseases, patients with chronic HBV require ongoing reassessment and engagement to determine if treatment is needed.^78^ This forces care into tertiary centres that might be less accessible to patients, which may result in falling follow-up and attendance rates over time.^78^ It is certain that the interruption to outpatient services from the COVID-19 pandemic will result in additional patients being lost to follow-up with those unfamiliar with the system likely to be at highest risk.

UK data has shown that it takes around 25 years for outpatient usage in a recently arrived migrant population to reach levels of a non-migrant population.^85^ Improved approaches are therefore needed to ensure patients remain under review within tertiary clinic settings. This includes greater education, as well as better integration of care. This could include shared care between GP practices in high prevalence areas with hepatology services or an outreach clinic model.^78^ Furthermore, educational resources and language translation services are comparatively poor for patients from SSA. Task shifting follow-up to community teams could improve engagement with patients, as attendance at a tertiary centre would only be required for appointments requiring a decision about treatment.^78^ Certainly, with improved infrastructure surrounding telehealth implemented due to the COVID-19 pandemic, physical appointments at tertiary centres are no longer required in all cases, improving access to specialist care.

## Conclusion

Worldwide elimination of chronic HBV will not occur by 2030 unless an evidence-based approach to the management of patients from SSA is adopted by high-income countries and leading hepatology societies. With a growing migrant population from SSA living in high-income countries, we have an opportunity to engage this marginalised group with chronic HBV. Evidence-based HCC guidelines must be developed, starting with validation of the current risk scores in this population. Hepatitis B publications must accurately define ethnicity. Collaboration should occur amongst centres to provide ‘real-world’ data to guide our current practice.

Local health services should create new opportunities for access to chronic HBV diagnosis and treatment for affected patients. Integration with HIV services may increase detection and enable sharing of resources and equipment. In lieu of a screening programme, outreach mobile community testing can be instituted, ideally utilising point of care diagnostics in areas of high prevalence. Integration of health technology can link antenatal screening results with appropriate providers for third trimester prophylaxis and labour management without stigmatisation. Post-COVID acceptance of telehealth can ensure specialist care is available to a wider population with increased flexibility.

Finally, truly global representation of chronic HBV must become the paradigm for clinical trials. Inclusion criteria should be reviewed to ensure patients from SSA are not arbitrarily excluded based on physiological laboratory values. Migrant communities should be engaged and educated about chronic HBV and the value of participating in trials. Clinical trials should target centres with a diverse ethnic population, or at least ensure adequate representation of genotypes and ethnicities in the study design. Participation by patients from SSA in trials in high-income regions will improve awareness of the public health importance of chronic HBV. With active engagement of this population in the search for a cure we will de-stigmatise chronic HBV and draw closer to achieving global elimination.

## Financial support

The authors received no financial support to produce this manuscript.

## Authors’ contributions

TM – conceptualisation, literature search, writing - original draft. SN – conceptualisation, literature search, writing - original draft. GD – supervision, writing - review and editing. KA – supervision, writing - review and editing.

## Conflict of interest

We have no conflict of interest to declare.

Please refer to the accompanying ICMJE disclosure forms for further details.
